# Supramolecular free radicals: near-infrared organic materials with enhanced photothermal conversion†
†Electronic supplementary information (ESI) available. See DOI: 10.1039/c5sc01167a


**DOI:** 10.1039/c5sc01167a

**Published:** 2015-04-20

**Authors:** Yang Jiao, Kai Liu, Guangtong Wang, Yapei Wang, Xi Zhang

**Affiliations:** a The Key Lab of Organic Optoelectronics & Molecular Engineering , Department of Chemistry , Tsinghua University , Beijing 100084 , P. R. China . Email: xi@mail.tsinghua.edu.cn; b Department of Chemistry , Renmin University of China , Beijing , 100872 , P. R. China

## Abstract

A novel kind of supramolecular free radical with significantly improved free radical yield and enhanced near-infrared (NIR) photothermal conversion has been fabricated. Perylene diimide (PDI) can undergo chemical reduction to generate PDI radical anions. Cucurbit[7]uril (CB[7]), a bulky hydrophilic head, was utilized to encapsulate the two end groups of the PDI derivative *via* host–guest interactions, thus hindering its aggregation and suppressing the dimerization and quenching of PDI radical anions in aqueous solution. Due to the increased concentration of radical anions and their absorption above 800 nm, the efficiency of NIR photothermal conversion was significantly improved. Compared with free radicals fabricated by covalent chemistry, the supramolecular free radicals established here could provide a facile approach for the promoted formation of aromatic free radicals, thus opening up a new strategy for the design of NIR photothermal materials with enhanced photothermal conversion.

## Abstract 2

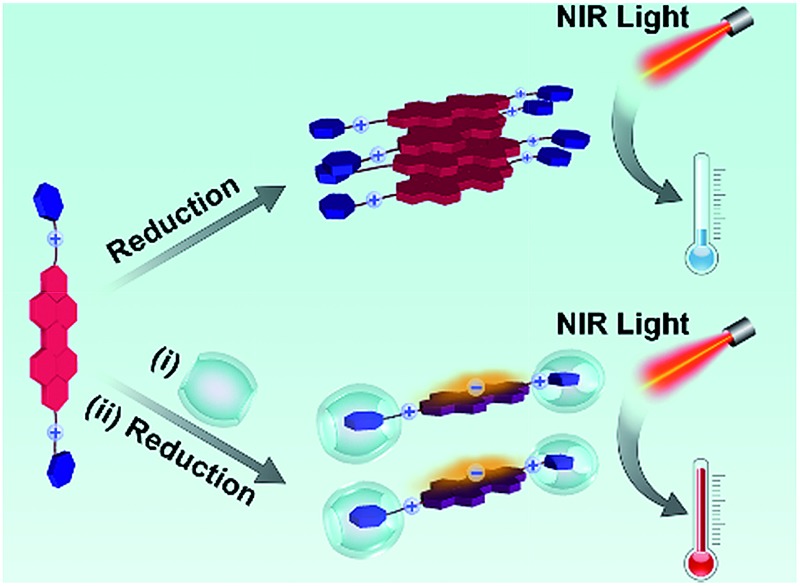
A novel kind of supramolecular free radical with significantly improved free radical yield and enhanced near-infrared photothermal conversion has been fabricated.

## Introduction

Photothermal materials have various applications, especially in photothermal therapy^1^ and light-triggered drug release.^2^ For most applications, they usually have a strong absorption in the near-infrared (NIR) region, as the region from 780 nm to 1300 nm, the so-called “biological window”, is where most biological tissues are highly transparent.^3^ Thus, NIR light is a preferred source to penetrate biological tissues and realize photothermal conversion *in vivo*. Among the promising candidates for photothermal materials are organic dyes, which absorb NIR light and convert the photo-energy into heat *via* non-radiative relaxation pathways such as molecular vibrations.^4^ Organic dyes not only exhibit considerable heat generation capabilities, but also have several advantages including easy accessibility, stability and potential flexibility for structure design and tunable applications. In the search for more organic systems with NIR absorption, we observed that delocalized radical anions could be generated from electron-deficient aromatic diimides under chemical or electrochemical reduction, inducing a red shift in the absorption.^5^ Among these, perylene diimide (PDI) radical anions have a typical red shift towards the NIR region.5c,d The inherent reactivity of radical anions and the aggregation of PDI^6^ usually cause PDI radical anions to dimerize and quench in aqueous environments, leading to a loss of free radical yield.^7^ Many efforts have been devoted to suppressing the quenching effect by covalently introducing bulky moieties onto the PDI cores,^8^ so that an improvement in PDI radical anion yield is achieved in non-aqueous solvents. To the best of our knowledge, the promoted formation of PDI radical anions by a supramolecular strategy in aqueous solution has not yet been reported.

**Scheme 1:**
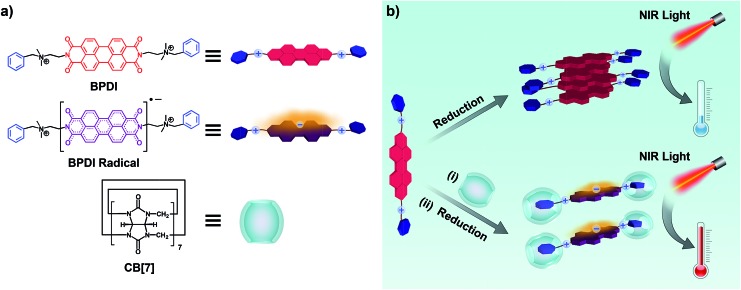
(a) Chemical structures of BPDI, BPDI radical anion and CB[7]. (b) BPDI radical anions generated by chemical reduction, leading to low NIR photothermal conversion efficiency (top); fabrication of the BPDI/(CB[7])_2_ supramolecular complex through host–guest interactions between CB[7] and the two benzyl end groups of BPDI, and the chemical reduction of this supramolecular complex, leading to the promoted formation of supramolecular free radicals with improved NIR photothermal conversion efficiency (bottom).

Cucurbit[*n*]urils (CB[*n*]), a family of barrel-shaped macrocyclic hosts, have attracted widespread attention in supramolecular chemistry^9^ and other related areas^10^ because of their exceptionally firm binding with cationic species. Due to their large molecular sizes and hydrophilic exteriors, CB[*n*] have been utilized as non-covalent steric hindrance blocks to optimize molecular properties.^11^ In this study, we have employed CB[7] in an attempt to weaken the close stacking of PDI aromatic cores, suppressing the dimerization and quenching of PDI radical anions, and thus improving the free radical yield in aqueous solution. As shown in Scheme 1, a bola-form amphiphile (BPDI) containing PDI as a rigid core was designed and synthesized. The other building block, CB[7], with a suitable cavity for the benzyl moiety, was expected to encapsulate the two end groups of BPDI through host–guest interactions, leading to the construction of a “dumbbell-shape” supramolecular complex. As expected, a novel kind of supramolecular free radical was generated *via* the reduction of the supramolecular complex, and the production of PDI radical anions could be markedly enhanced by the steric hindrance of CB[7]. In addition, the introduction of two quaternary ammonium groups in the BPDI molecule and the encapsulation of CB[7] make the system water-soluble. It is conceivable that the supramolecular free radicals, which could be fabricated by a simple and facile process, have enough solubility and stability in water for subsequent photothermal conversion. Finally, the NIR photothermal conversion efficiency could be promoted by the increased concentration of PDI radical anions with absorption above 800 nm.

## Results and discussion

**Fig. 1:**
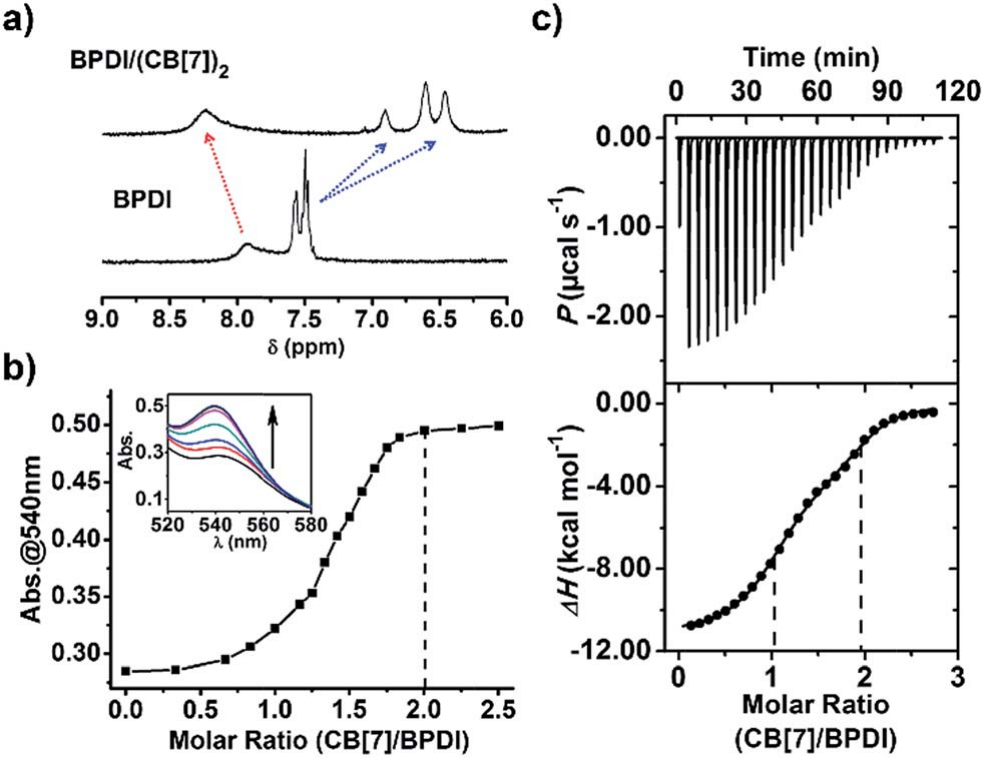
(a) Partial ^1^H NMR spectra of BPDI (0.5 mM) and BPDI/(CB[7])_2_ (0.5 mM) aqueous solutions. (b) UV-Vis titration results of absorbance at 540 nm against the molar ratio of CB[7] : BPDI. Inset: typical UV-Vis spectral changes of BPDI with the addition of CB[7] in aqueous solution. (c) ITC data and fitting curves of CB[7] titrated into BPDI. *c*(BPDI) = 0.05 mM, *c*(CB[7]) = 0.65 mM.

Various methods including NMR, fluorescence spectroscopy, UV-Vis spectroscopy and isothermal titration calorimetry (ITC) were employed to confirm the complexation between BPDI and CB[7] in aqueous solution. As shown in the ^1^H NMR spectra, upon addition of 2 equivalents of CB[7] to BPDI solution, the benzyl proton peaks of BPDI (around 7.5 ppm) shifted upfield as a consequence of CB[7] inclusion. Concurrently, the PDI proton peaks (around 7.9 ppm) shifted downfield because of the weakening of π–π stacking (Fig. 1a). This indicates the formation of a host–guest supramolecular complex by the encapsulation of the two benzyl end groups of BPDI with CB[7]. Moreover, as indicated in Fig. S1,† the π–π stacking between adjacent PDI chromophores caused a significant quenching of fluorescence,11*c* while after the addition of CB[7], a recovery of fluorescence could be observed, suggesting the deaggregating effect of the supramolecular complex.

UV-Vis spectroscopic titration experiments were performed to quantitatively study the complexation. The concentration of BPDI was fixed at 0.3 mM, and with the addition of CB[7], the π–π stacking between the adjacent PDI chromophores weakened, causing a gradual increase in the characteristic absorption at 540 nm (Fig. 1b inset). As shown in Fig. 1b, by analysing the absorbance change at 540 nm at different molar ratios, we found that the absorbance nearly stopped increasing after the molar ratio reached 2. Therefore, the binding stoichiometry between BPDI and CB[7] was determined to be 1 : 2.

We also used ITC to collect more thermodynamic information about the complexation. From the obtained titration data shown in Fig. 1c, two binding sites could be observed, and the second site corresponded to a molar ratio of 1 : 2. This indicates that one BPDI molecule with two benzyl end groups can be associated with two CB[7] molecules, which is consistent with the results of the UV-Vis spectroscopic titration. By fitting the data, the binding constant of BPDI with CB[7] was calculated to be 2.9 × 10^12^ M^–2^. Such strong interactions result from a combination of host–guest interactions and electrostatic attraction between the quaternary ammonium groups in BPDI and the seven carbonyl groups at the edge of CB[7]. Therefore, the host–guest interactions are strong enough for the construction of the BPDI/(CB[7])_2_ supramolecular complex with a well-defined composition.

**Fig. 2:**
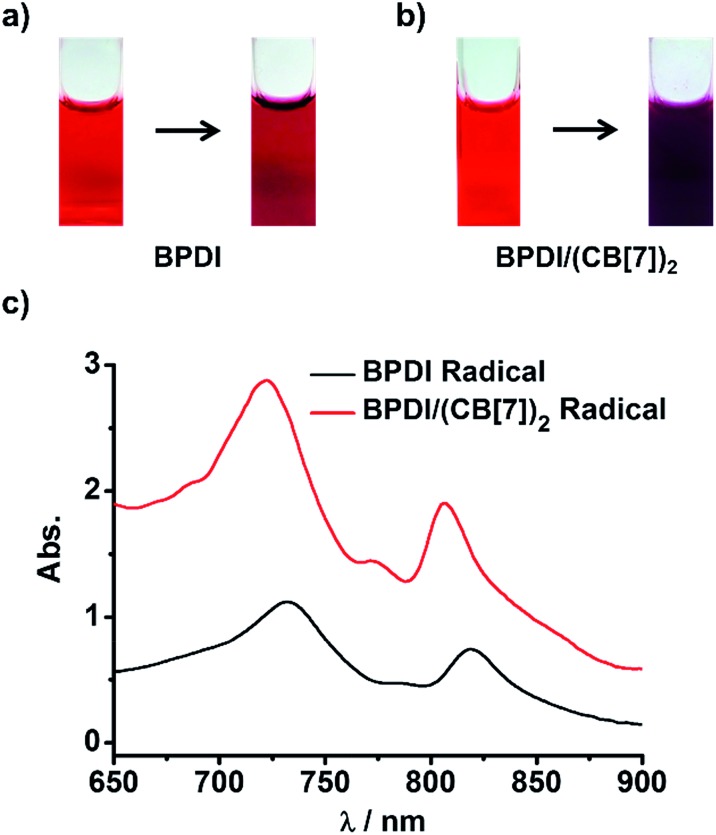
Photographs of the process of radical anion generation from (a) BPDI (0.3 mM) and (b) BPDI/(CB[7])_2_ (0.3 mM). (c) UV-Vis spectra of the solutions containing PDI radical anions generated from BPDI and BPDI/(CB[7])_2_.

We wondered if the BPDI/(CB[7])_2_ supramolecular complex could generate more PDI radical anions than BPDI itself under the same conditions. To answer this question, sodium dithionite (Na_2_S_2_O_4_) was selected to reduce BPDI and BPDI/(CB[7])_2_ in aqueous solution to produce PDI radical anions. In a typical process, Na_2_S_2_O_4_ solution (in pH 8 borate buffer) was freshly prepared and then injected into a sealed cuvette containing BPDI aqueous solution after a constant bubbling of nitrogen gas for 30 min.7*a* After injection of Na_2_S_2_O_4_ with stirring, the color of the BPDI solution immediately changed from red to purplish-red (Fig. 2a), which could be distinguished by the naked eye, suggesting the production of PDI radical anions. UV-Vis spectroscopy provided quantitative evidence. The characteristic absorption bands of PDI peaking at 500 nm and 540 nm decreased in intensity, while characteristic absorption bands of PDI radical anions appeared at 732 nm and 819 nm (Fig. S2†). Therefore, it was demonstrated that the reduction of BPDI with Na_2_S_2_O_4_ could produce PDI radical anions. At the same concentration, BPDI/(CB[7])_2_ solution could generate a significant amount of PDI radical anions after the same treatment. As shown in Fig. 2b, the color of the reduced BPDI/(CB[7])_2_ solution was dark purple—much darker than that of the reduced BPDI solution. Accordingly, the characteristic absorption of PDI radical anions appeared in the same region but with a clear increase in the absorbance (Fig. 2c), indicating that the amount of PDI radical anions generated by the BPDI/(CB[7])_2_ solution was twice as many as the amount produced by BPDI alone.

**Fig. 3:**
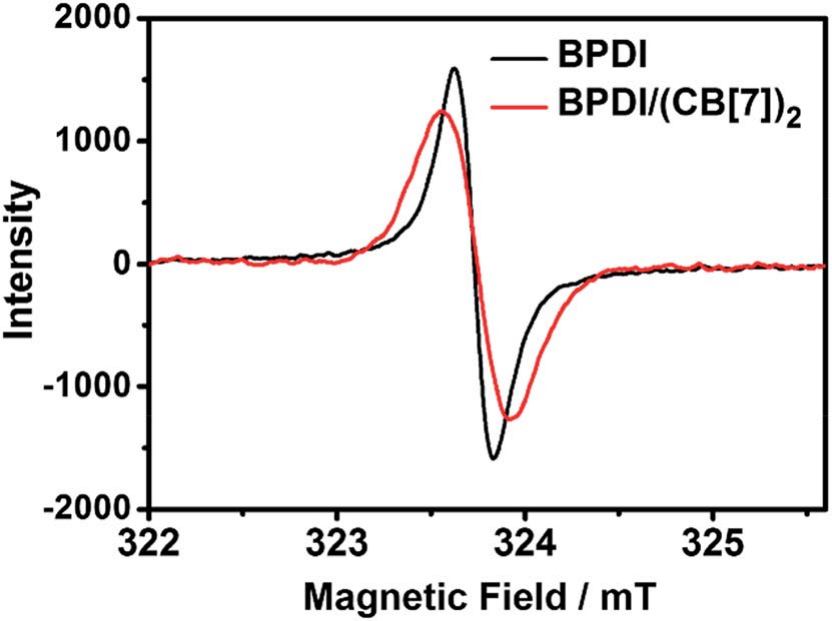
EPR spectra of the radical anions generated from BPDI (0.3 mM) and BPDI/(CB[7])_2_ (0.3 mM).

In order to provide direct evidence for the formation of PDI radical anions, electron paramagnetic resonance (EPR) spectroscopy was also carried out for the two solutions after reduction. As shown in Fig. 3, they both displayed a typical EPR signal, proving the existence of free radicals. Upon further analysis of the EPR signals, some important parameters could be obtained (Table 1). The *g*-factor for both BPDI and BPDI/(CB[7])_2_ radical anions was found to be *g* = 2.0035, which is consistent with previously reported values,8a,^12^ confirming the formation of PDI radical anions. The same *g* value indicates that the two radical anions have similar structures. The integration of the EPR signals was calculated to be 14.7 for BPDI radical anions and 30.3 for BPDI/(CB[7])_2_ radical anions. Through the EPR standard curve (Fig. S4†), the concentration of BPDI radical anions was determined to be 0.016 mM, and that of BPDI/(CB[7])_2_ radical anions was 0.033 mM. Therefore, the concentration of BPDI/(CB[7])_2_ radical anions was twice that of the BPDI radical anions, which is consistent with the above UV-Vis data.

**Table 1:** Detailed EPR signal parameters and radical anion concentrations calculated from the integration values

	BPDI radical anion	BPDI/(CB[7])_2_ radical anion
*g*-Factor	2.0035	2.0035
Integration	14.7	30.3
Radical anion concentration (mM)	0.016	0.033

**Fig. 4:**
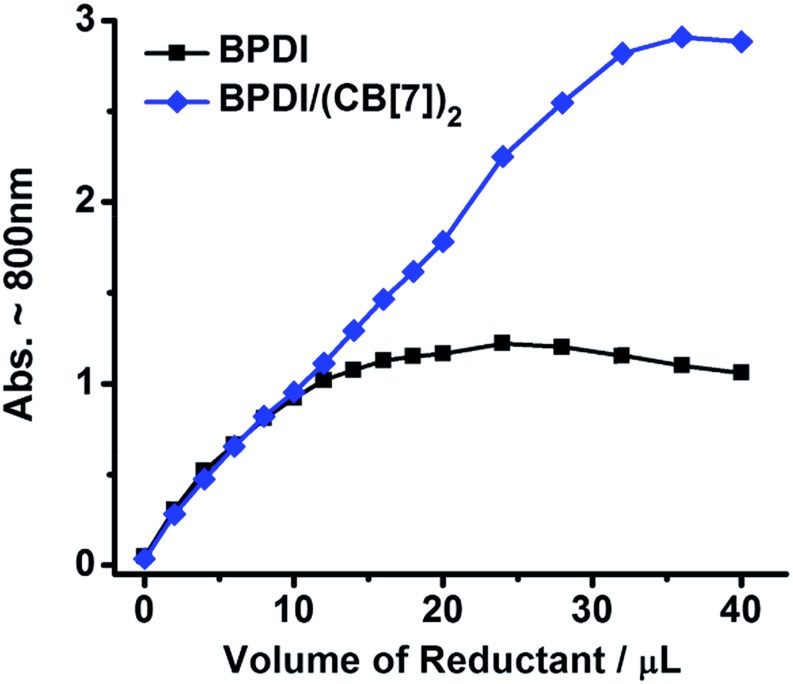
The changing amount of radical anions generated with an increasing amount of reductant (BPDI or BPDI/(CB[7])_2_ solution (0.3 mM, 2 mL) was reduced by 30 mM Na_2_S_2_O_4_ solution).

To comprehensively compare the free radical yields, a reduction titration with gradual addition of the reductant to BPDI or BPDI/(CB[7])_2_ solution was performed and monitored by UV-Vis spectroscopy. The absorbance for the characteristic band around 800 nm, which could reflect the amount of radical anions generated in the process, was plotted *versus* the volume of the Na_2_S_2_O_4_ solution. As shown in Fig. 4, at the beginning of the titration, only a small amount of radical anions were produced, and the low radical concentration was not conducive to the dimerization and quenching of radical anions, thus the free radical yields of the two solutions were nearly identical because the steric effect of CB[7] was not significant at such a low concentration. However, with an increasing dosage of reductant, the concentration of radical anions rose gradually and the two samples performed very differently. BPDI radical anions easily dimerized at higher concentrations,7a,^13^ causing a stagnation in the increase of absorbance. In the BPDI/(CB[7])_2_ solution, however, the bulky CB[7] hindered the aggregation of adjacent radical anions. Consequently, the free radical yield in the BPDI/(CB[7])_2_ solution continued to rise. Therefore, the steric hindrance of CB[7] blocks is critical to the improved free radical yield of supramolecular free radicals.

**Fig. 5:**
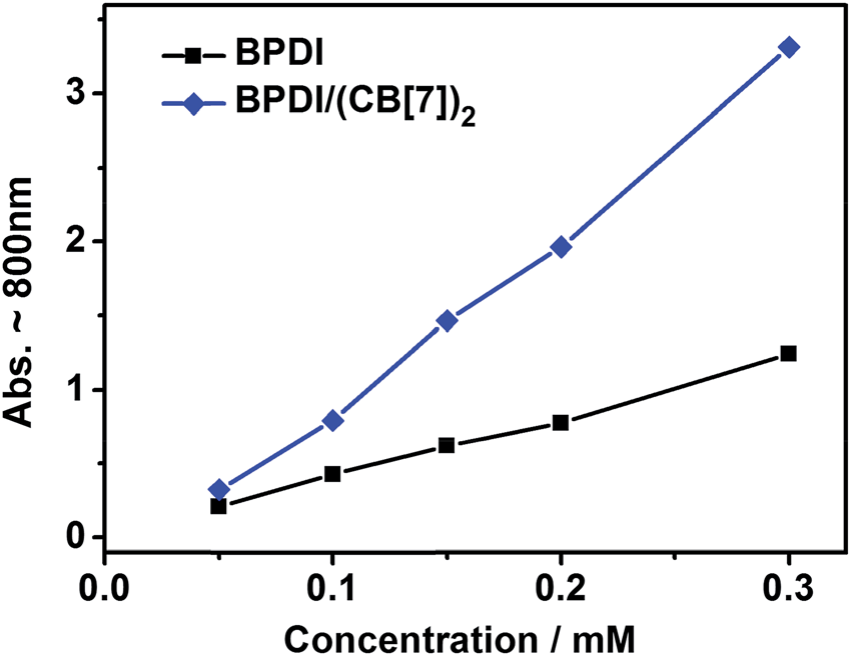
The maximum free radical yields (monitored by UV-Vis) in BPDI and BPDI/(CB[7])_2_ solutions at different concentrations. The series of concentrations are 0.05, 0.1, 0.15, 0.2 and 0.3 mM.

To further understand the mechanism of the improvement in free radical yield, we carried out reduction of the two solutions at different concentrations and then measured the free radical yield by UV-Vis spectroscopy. As shown in Fig. 5, the maximum free radical yields for the two solutions both increased with increasing concentration, as a consequence of the increased amount of PDI moieties. Remarkably, with the increase in concentration, the maximum yield of BPDI/(CB[7])_2_ supramolecular free radical increased much faster than that of the BPDI radical anion, which could also be demonstrated by calculating the improvement ratio of the BPDI/(CB[7])_2_ supramolecular free radical yield against the BPDI radical anion (Fig. S6†). As mentioned above, after the formation of the supramolecular complex, the bulky CB[7] heads suppress the aggregation of BPDI. Therefore, at higher concentration, as a result of the more intense aggregation of BPDI, the deaggregating effect of CB[7] is more significant, inducing a greater improvement in the free radical yield. This phenomenon further supports the mechanism described above.

**Fig. 6:**
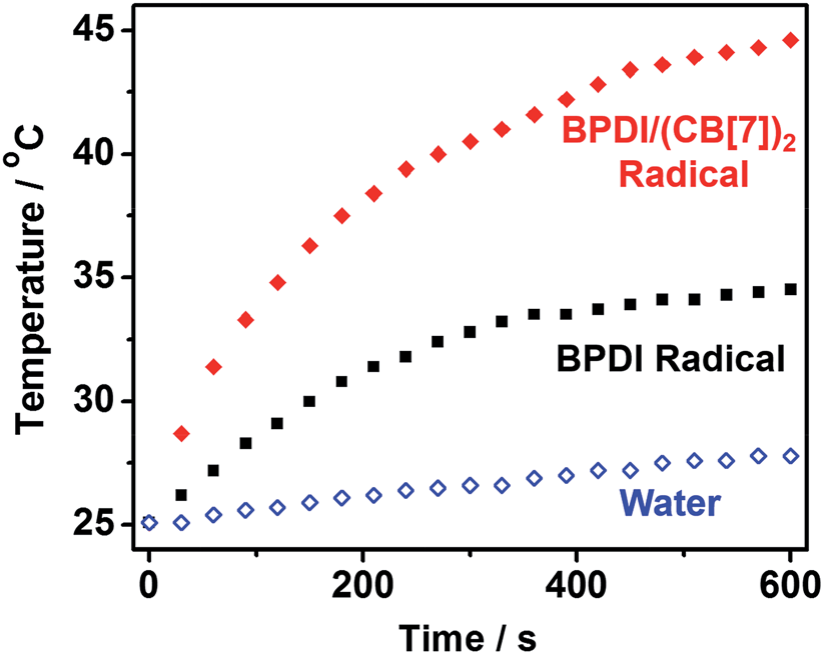
Photothermal conversion experimental data: temperature elevation of aqueous solutions containing BPDI radical anions or BPDI/(CB[7])_2_ radical anions as a function of time (0–600 s) under irradiation by an 808 nm laser at a power density of 1.00 W cm^–2^. Pure water was used as the blank control.

Having fabricated the BPDI/(CB[7])_2_ supramolecular free radicals and improved the PDI free radical yield using CB[7] blocks, we anticipated that the supramolecular free radical could attain a more effective NIR photothermal conversion. Hence, we performed photothermal conversion experiments. With 808 nm irradiation at 1 W cm^–2^ and room temperature (25.1 °C), the temperature elevation of aqueous solutions containing BPDI radical anions or BPDI/(CB[7])_2_ radical anions was measured (Fig. 6). A blank test demonstrated that the temperature of pure water increased by less than 3 °C within 10 min. On the other hand, a significant increase in temperature was observed after irradiating BPDI radical anions or BPDI/(CB[7])_2_ radical anions. It should be noted that the solution containing BPDI/(CB[7])_2_ supramolecular free radicals had a faster rate as well as a greater temperature increase than the solution containing BPDI radical anions. Within 10 min, the temperature of the BPDI solution increased by 9.4 °C, whereas that of the BPDI/(CB[7])_2_ solution increased by 19.5 °C. Thus, the 808 nm laser heat conversion efficiency was determined to be 16.3% for BPDI radical anions and 31.6% for BPDI/(CB[7])_2_ radical anions, according to the modified calculation method used for photothermal conversion efficiency by Roper *et al.*^14^ (see Fig. S7 and Table S1 in the ESI†). It is evident that the NIR photothermal conversion efficiency of the supramolecular free radicals was enhanced by approximately 94% compared to that of their building blocks.

In addition to the facile fabrication using this supramolecular strategy, one more advantage of supramolecular free radicals is the reversibility inherent from the dynamic host–guest interactions. 1-Adamantanamine hydrochloride (AD), which has a binding constant as high as 4.2 × 10^12^ M^–1^ with CB[7],9*d* was added to the BPDI/(CB[7])_2_ supramolecular complex. The preferred binding of CB[7] to the AD guest led to the dissociation of the BPDI/(CB[7])_2_ supramolecular complex and the recovery of the close stacking of PDI aromatic cores, as confirmed by ^1^H NMR, UV-Vis and fluorescence spectroscopy (Fig. S8†). The spectra of BPDI/(CB[7])_2_/AD_2.5_ were nearly the same as those for BPDI itself. In addition, the radical anions generated in BPDI/(CB[7])_2_/AD_2.5_ solution recovered to the initial quenched state without the assistance of CB[7] heads (Fig. S8†). All the above results revealed that supramolecular free radicals are highly reversible and adaptive.

## Conclusions

In summary, we have successfully fabricated a novel kind of supramolecular free radical with significantly improved free radical yield and NIR photothermal conversion efficiency. The fabrication of the supramolecular free radical is based on host–guest interactions, and is facile, reversible and highly efficient. The supramolecular free radical has an improved photothermal conversion efficiency (31.6%) compared to commercial gold nanorods (∼22%),1c,d which are one of the most widely known photothermal systems. Furthermore, the dynamic and adaptive properties derived from supramolecular interactions exhibit potential for the construction of smart materials. We anticipate that this supramolecular approach could be extended to other aromatic free radicals, and supramolecular free radicals with enhanced photothermal conversion may open up applications in photothermal therapy and light-triggered drug release.

## Supplementary Material

Supplementary information
