# Supplementary material for: Supramolecular free radicals: near-infrared organic materials with enhanced photothermal conversion
Source: Chem Sci. 2015 Apr 20;6(7):3975–80. doi: 10.1039/c5sc01167a (PMC5707502; doi:10.1039/c5sc01167a)
Supplement: Supplementary file 1 [file SC-006-C5SC01167A-s001.pdf]

Electronic Supplementary Material (ESI) for Chemical Science.
This journal is © The Royal Society of Chemistry 2015

Supramolecular Free Radicals: Near-infrared Organic Materials with Enhanced Photothermal Conversion

*Yang Jiao, Kai Liu, Guangtong Wang, Yapei Wang and Xi Zhang**

Supporting Information

1. General instrumentation and methods.

The NMR spectra were obtained using a JOEL JNM-ECA400 apparatus.

The UV-Vis spectra were measured using a HITACHI U-3010 spectrophotometer. For the experiments in Figure 1b and Figure S8b, path length was 0.5 mm. While for the other UV-Vis spectra, path length was 10 mm.

The fluorescence spectra were measured using a HITACHI F-7000 apparatus. The excitation wavelength was 550 nm, slit: 20.0 nm, scanning rate: 240 nm/min.

Electronic paramagnetic resonance (EPR) measurements were performed on a JEOL JES-FA200 apparatus, with 9055 MHz microwave frequency and 0.998 mW power. TEMPO was selected for the measurement of EPR standard curve and quantitative calculation of radical anion concentrations.

Isothermal titration calorimetry (ITC) experiments were carried out with a Microcal VP-ITC apparatus in acetate buffer solution (pH 4.75) at 298.15 K.

Photothermal conversion experiments were performed with the following process: $Na_2S_2O_4$ buffer solution (15 mM) was prepared freshly and injected with a 40 μL dosage into BPDI or BPDI/$(CB[7])_2$ aqueous solution (0.3 mM, 1 mL) after a constant bubbling of nitrogen gas for 30 min. After the formation of PDI radical anions, the 808 nm laser was employed to irradiate the solution at a power density (1.00 W cm^{-2}), and the temperature elevation of the solution was measured in real time. Similar experiments was also carried out for pure water as the blank control, and for BPDI or BPDI/$(CB[7])_2$ solution without reduction to achieve a more accurate calculation of photothermal conversion efficiency.

2. Synthesis of BPDI.

The synthetic route is shown in Scheme S1. 3,4,9,10-perylenetetracarboxylic dianhydride (0.5 g) was dissolved in N,N-Dimethyl-1,2-ethanediamine (15 mL). The solution was refluxed with stirring overnight. The solution was allowed to cool and 50-100 mL methanol was added to the mixture, which was then boiled for 30 min. The dark purple solid precipitate (Compound 1) was filtered and washed with methanol then dried under vacuum. The yield was 90 %.

^{1}H NMR (400MHz, CF_3COOD): 8.75~8.95 (m, 8H), 4.81 (s, 4H), 3.78 (s, 4H), 3.22 (s, 12H).

0.5 g Compound 1 and 5.0 mL benzyl bromide were mixed in 20 mL DMF. The resulting mixture was then stirred at 80°C for 5 h. The solution was added dropwise into 150 mL of toluene. The dark red solid precipitate (BPDI) was filtered and washed by toluene. The yield was 95 %.

^{1}H NMR (400MHz, DMSO-d_6): 8.79 (d, J = 8.0Hz, 6H), 8.45 (d, J = 8.0Hz, 4H), 7.40~7.52 (m, 8H), 4.50 (s, 4H), 4.08~4.22 (m, 4H), 3.34~3.48 (m, 4H), 2.93 (s, 12H).

ESI-MS: m/z = 357.16 (Calculated for $C_{46}H_{42}N_4O_4^{2+}$: m/z = 357.18)

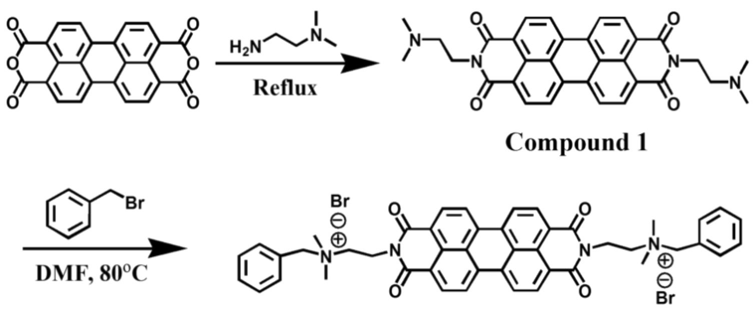

Scheme S1. Synthetic route of BPDI.

3. Fluorescence emission spectra of BPDI and BPDI/(CB[7])$_2$ aqueous solutions.

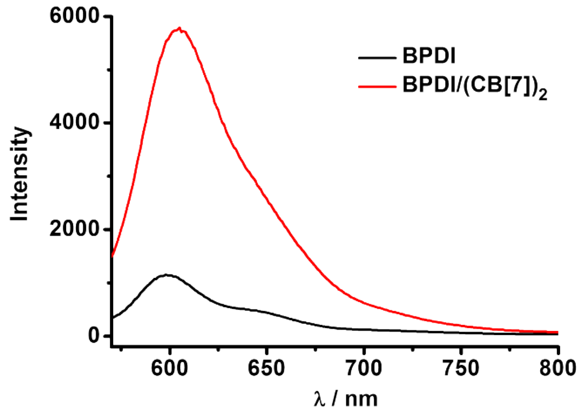

Figure S1. Fluorescence emission spectra of BPDI (0.3 mM) and BPDI/(CB[7])$_2$ (0.3 mM) in aqueous solutions.

4. Full UV-Vis spectra of BPDI and BPDI radical anion in aqueous solution.

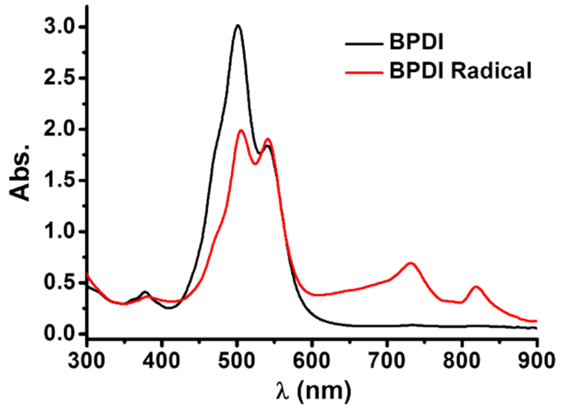

Figure S2. Full UV-Vis Spectra of BPDI and BPDI radical anion in aqueous solution. The characteristic absorption bands peaked at 500 nm and 540 nm decreased accompanied with the increase of absorbances at 732 nm and 819 nm, indicating the consuming of BPDI to produce BPDI radical anions.

5. The non-fluorescent properties of BPDI radical anions and BPDI/(CB[7])$_2$ radical anions.

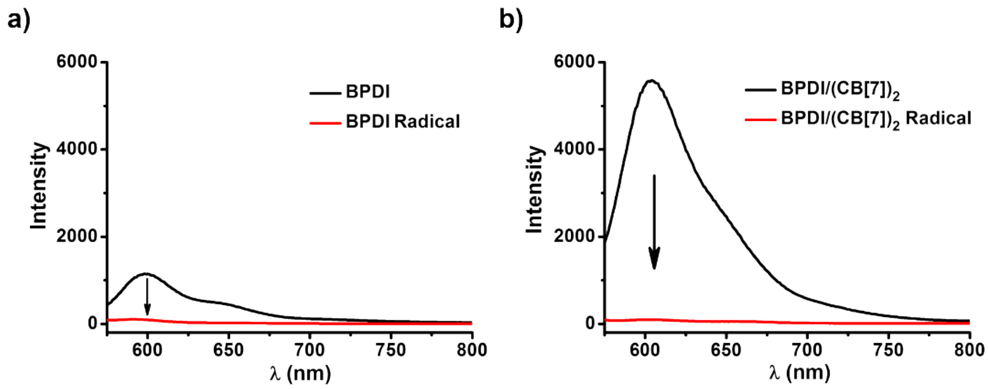

Figure S3. Fluorescence emission spectra of a) BPDI and BPDI radical anion, b) BPDI/(CB[7])$_2$ and BPDI/(CB[7])$_2$ radical anion. After the formation of radical anions, the fluorescence disappeared completely for either solution.

6. Quantitative calculation for the concentration of BPDI radical anions and BPDI/(CB[7])$_2$ radical anions.

By further analysing the EPR signals, the integration of BPDI radical anions and BPDI/(CB[7])$_2$ radical anions could be determined. Then we could calculate the radical anion concentrations from the integration on the basis of the standard curve (Figure S4).

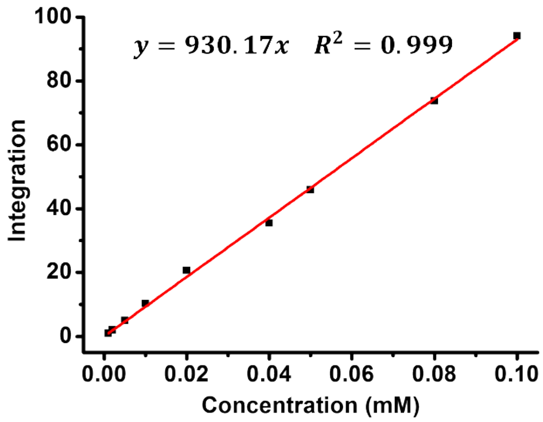

Figure S4. EPR standard curve for quantitative calculation of free radical concentration. The curve was measured by performing the EPR experiments for TEMPO standard solutions at a series of concentrations (0.001 mM, 0.002 mM, 0.005 mM, 0.01 mM, 0.02 mM, 0.04 mM, 0.05 mM, 0.08 mM and 0.1 mM).

7. The process of reduction titration represented by the evolution of UV-Vis Spectra of BPDI or BPDI/(CB[7])$_2$ solution.

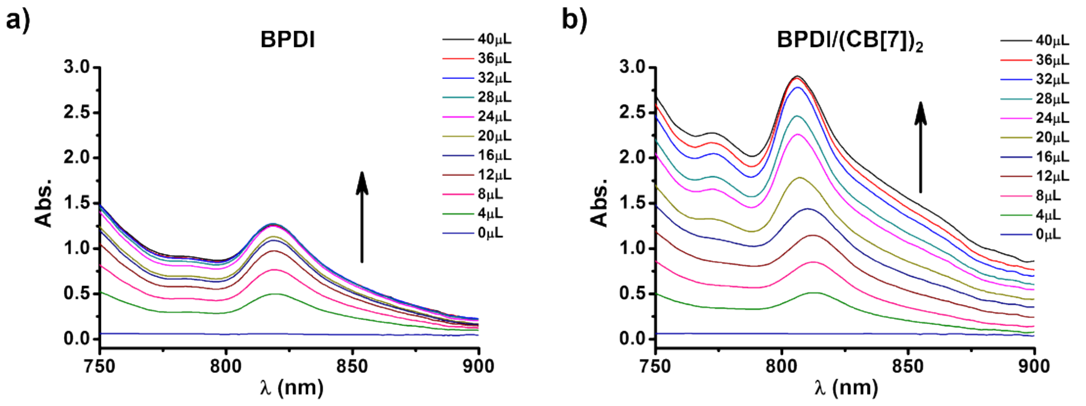

Figure S5. Typical UV-Vis spectral changes during the reduction titration with $Na_2S_2O_4$ solution into a) BPDI and b) BPDI/(CB[7])$_2$ aqueous solution. The concentrations and usages of BPDI and BPDI/(CB[7])$_2$ are both fixed at 0.3 mM and 2 mL, respectively. c($Na_2S_2O_4$) = 30 mM (dissolved in pH 8 borate buffer).

8. Improvement ratio of BPDI/(CB[7])$_2$ supramolecular free radical yield against BPDI radical anion at different concentrations.

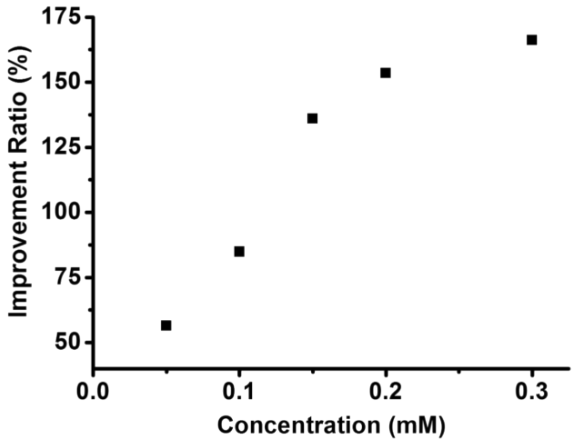

Figure S6. Improvement ratio of BPDI/(CB[7])$_2$ supramolecular free radical yield against BPDI radical anion at different concentrations. The series of concentrations of BPDI or BPDI/(CB[7])$_2$ are 0.05, 0.1, 0.15, 0.2, 0.3 mM.

Improvement ratio is defined as follow:

$$Improvement\ ratio = \frac{Y(BPDICB[7]) - Y(BPDI)}{Y(BPDI)} \times 100\ \%$$

where *Y*(BPDI) is the maximal yield of BPDI radical anion, and *Y*(BPDICB[7]) is the maximal yield of BPDI/(CB[7])$_2$ radical anion.

9. Stability of supramolecular free radicals.

The stability of the supramolecular free radical was measured at 25°C and 60°C, respectively. The BPDI/(CB[7])$_2$ supramolecular free radical remains 90% after 1 h at 25°C. Moreover, the supramolecular free radical also has a good stability even at 60°C. Considering that the photothermal conversion process is usually completed within 30 min, such stability of the supramolecular free radical should be enough for the photothermal conversion.

10. The detailed derivation and calculation of photothermal conversion efficiency for BPDI radical anion and BPDI/(CB[7])$_2$ radical anion.

The photothermal conversion efficiency could be calculated from the energy balance during the irradiation for BPDI or BPDI/(CB[7])$_2$ solution. The total energy balance for the system is:

$$mC_p\frac{dT}{dt} = Q_{in,radical} + Q_{in,surr} - Q_{out} \quad (1)$$

where m and C_p are the mass and heat capacity of the solvent (water) and T is the solution temperature. $Q_{in,radical}$ is the photothermal energy input from the radical anions. Define the photothermal conversion efficiency η as the fraction of the total light energy that is converted to heat, that is:

$$\eta = \frac{Q_{in,radical}}{I}$$

where I is the laser power. $Q_{in,surr}$ is the heat generation from the BPDI or BPDI/(CB[7])$_2$ solution, while Q_{out} is the heat loss to the surroundings, following the theory of convection heat transfer:

$$Q_{out} = hA(T - T_{surr}) \quad (2)$$

Where h is the heat transfer coefficient, A is the irradiated area of the container and T_{surr} is the temperature of surrounding (can be regarded as constant). When the system reaches at the steady state, the rate of photothermal heating is equal to the rate of heat dissipation to the surrounding, thus achieving the energy balance and the maximal temperature, so that:

$$Q_{in,radical} + Q_{in,surr} = Q_{in} = hA(T_{max} - T \quad (3)$$

From the equation (2) and (3) the equation (1) will become to be:

$$mC_p\frac{dT}{dt} = hA(T_{max} - T)$$

A characteristic rate constant has been defined as $\tau_{out} = mC_p/hA$, the above differential equation can be solved:

$$\frac{dT}{T_{max} - T} = \frac{dt}{\tau_{out}}$$

Integrating using the initial condition, we can get:

$$\ln\left(\frac{T_{max} - T}{T_{max} - T_{surr}}\right) = -\frac{t}{\tau_{out}}$$

θ, a dimensionless driving force of temperature is defined as follow:

$$\theta = \frac{T - T_{surr}}{T_{max} - T_{surr}}$$

By this way the equation has been simplified as this:

$$\ln(1 - \theta) = -\frac{t}{\tau_{out}}$$

From the data of photothermal experiments, we can achieve the characteristic rate constant τ_{out} with the linear fitting method, as shown in Figure S7.

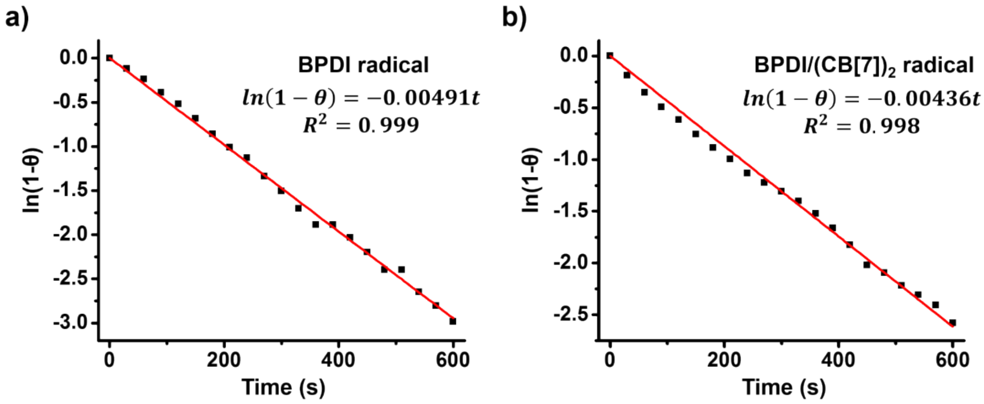

Figure S7. Linear fitting of temperature driving force θ to the irradiation time t to give the characteristic rate constant τ_{out} for a) BPDI radical anion and b) BPDI/(CB[7])$_2$ radical anion.

The characteristic rate constant τ_{out} of BPDI radical anion or BPDI/(CB[7])$_2$ radical anion can be attained from the fitting slopes, and then hA can also be determined, thus calculating the total heat generation:

$$Q_{in,radical} + Q_{in,surr} = Q_{in} = hA(T_{max} - T_{surr})$$

Similarly, $Q_{in,surr}$ can be calculated by dealing with the data for BPDI and BPDI/(CB[7])$_2$ solution without reduction. So far the photothermal conversion efficiency could be calculated by:

$$\eta = \frac{Q_{in,radical}}{I} = \frac{Q_{in} - Q_{in,surr}}{I}$$

	**BPDI Radical anion**	**BPDI/(CB[7])$_2$ Radical anion**
**Q_{in} (mW)**	192.9	355.4
**$Q_{in,surr}$ (mW)**	30.2	39.2
**$Q_{in,radical}$ (mW)**	162.7	316.2
**η (%)**	**16.27**	**31.62**

Table S1. Some important results calculated from the data of photothermal conversion experiments. The photothermal conversion efficiency η are marked in red for emphasis.

11. Adaptivity of supramolecular free radicals.

1-Adamantanamine hydrochloride (AD), can be used to disassociate the complexation between BPDI and CB[7], which has been confirmed by a variety of methods. As shown in Figure S8a, the evolution of 1H NMR induced by the inclusion of CB[7] recovered after the addition of AD. In addition, the separation of CB[7] heads led to the close aggregation of BPDI once again, resulting in the decrease of UV-Vis absorbance and the quenching of fluorescence emission (Figure S8b and S8c).

Moreover, the spectra of BPDI/(CB[7])$_2$/AD$_{2.5}$ were almost the same as those of BPDI, indicating the perfect reversibility and adaptivity of the supra-amphiphile. More importantly, the above dynamic properties have also been introduced into the supramolecular free radical. The production of PDI radical anions can be adjusted to the initial level (Figure S8d).

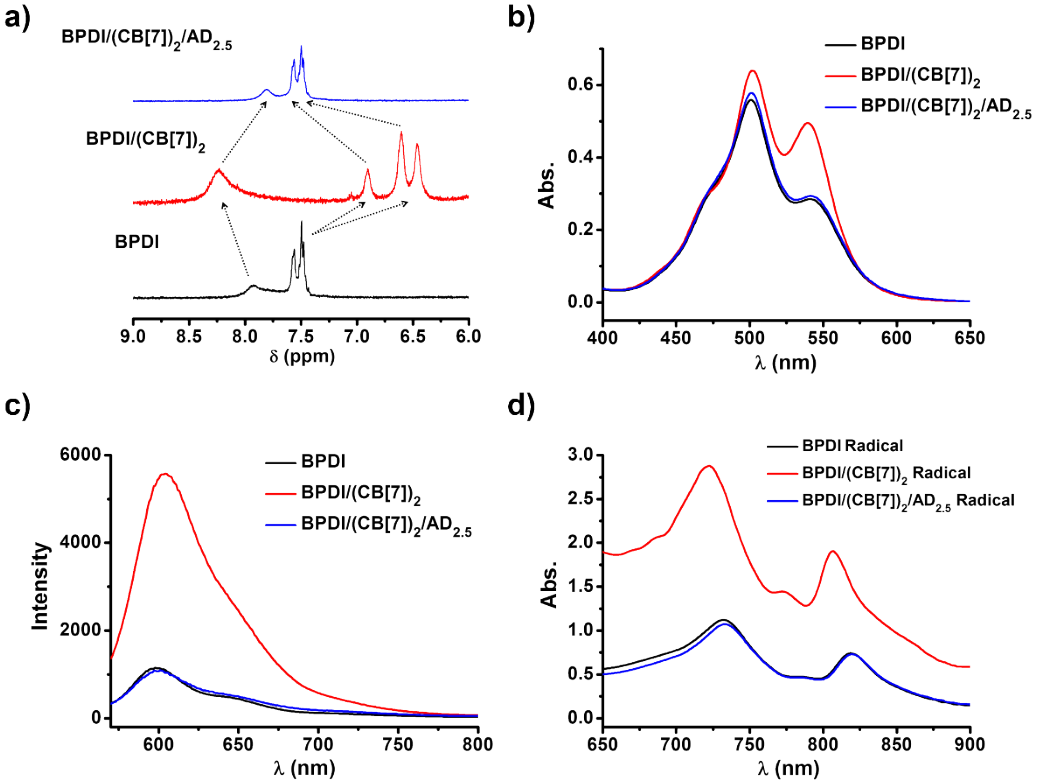

Figure S8. a) Partial ^{1}H NMR spectra, b) UV-Vis spectra and c) Fluorescence emission spectra of BPDI, BPDI/(CB[7])$_2$ and BPDI/(CB[7])$_2$/AD$_{2.5}$. d) UV-Vis spectra of the three kinds of radical anions generated from BPDI, BPDI/(CB[7])$_2$ and BPDI/(CB[7])$_2$/AD$_{2.5}$ in aqueous solutions.
